# Schistosomiasis in the Philippines: Innovative Control Approach is Needed if Elimination is the Goal

**DOI:** 10.3390/tropicalmed4020066

**Published:** 2019-04-13

**Authors:** Remigio M. Olveda, Darren J. Gray

**Affiliations:** 1 Research Institute for Tropical Medicine, Manila 1781, Philippines; 2 Research School of Population Health, Australian National University, Canberra 2601, Australia; darren.gray@anu.edu.au

**Keywords:** Schistosomiasis, Philippines, schistosomiasis elimination, *S. japonicum* zoonosis, bovines

## Abstract

In 1996, schistosomiasis due to *Schistosoma japonicum* was declared eradicated in Japan. In the People’s Republic of China, *S. japonicum* transmission has been interrupted in the major endemic areas in the coastal plains but the disease persists in the lake and marshland regions south of the Yangtze River. The disease remains a public health problem in endemic areas in the Philippines and in isolated areas in Indonesia. Comprehensive multidisciplinary campaigns had led to eradication of schistosomiasis in Japan and have been successful in the interruption of disease transmission in the major endemic regions of the People’s Republic of China. Unfortunately, the integrated measures cannot be duplicated in schistosomiasis endemic areas in the Philippines because of limited resources. The problem is also more complicated due to the topography in the Philippines and transmission is not seasonal as in China. An innovative approach is needed in the Philippines if schistosomiasis elimination is the goal.

## 1. Endemic Areas and Transmission of *Schistosoma japonicum* in the Philippines

Schistosomiasis remains a public health problem in endemic areas in the Philippines with approximately 12 million people residing in 28 endemic provinces located across 12 different geographical zones at risk of *S. japonicum* infection [1,2,3,4,5]. A total of 190 municipalities and 1212 barangays (villages) are currently endemic, based on surveys conducted over the past decade. Two new endemic foci reported in the northern (Gonzaga, Cagayan) and central (Calatrava, Negros Occidental) parts of the country were confirmed in 2004 and 2006, respectively [6]. Just like in China, bovines, water buffaloes (carabaos) in particular, play a major role in the transmission of schistosomiasis in the Philippines with infection prevalence close to 90% in some endemic barrangays. A 2011 study carried out in the municipality of Palapag, Northern Samar showed the *S. japonicum* prevalence in cattle to be 87.5% and 77.1% via real-time PCR (qPCR) and the formalin-ethyl acetate sedimentation (FEA-SD), respectively. In carabao, the *S. japonicum* prevalence was 79.1% and 55.2% by qCPR and FEA-SD, respectively [7]. The same study computed the Bovine Contamination Index (BCI) using the FEA-SD technique and gave an average of 195,000 eggs per bovine per day [7]. A recent study in Leyte Province showed a 97% prevalence via perfusion, 67.7% via qPCR and 34.3% by FEA-SD [8].

## 2. Effect of Long-term Infection with *Schistosoma japonicum*

Prolonged and repeated infection with *S. japonicum* cause two types of morbidities in schistosomiasis japonica: those with clear end-organ complications and those with subtle manifestations. Clear end-organ complications are due to granuloma and fibrosis formed around the parasite eggs trapped in the host tissues. The sequelae can be categorized into hepatosplenic, hepatointestinal, pulmonary, cerebral, and ectopic forms [9,10] Cardiac and renal localization of lesions are rarely encountered. Those with subtle manifestations are due to inflammatory cytokines induced by eggs or worm products of the parasite. Anaemia, growth retardation, malnutrition and impaired cognitive functions have been documented in children with *S. japonicum* infection [11,12,13,14]. Maternal schistosomiasis japonica has been shown to exert a negative impact on pregnancy outcomes. Babies born from mothers infected with *S. japonicum* have markedly decreased birth weight. Circulating mediators of inflammation are elevated in the peripheral blood, placental blood, and placental tissues of *S. japonicum* infected pregnant women. Placental TFN-α has also been associated with both *S. japonicum* infection and markedly decreased birth weight [15]. *S. japonicum* infection in pregnant women also result in up-regulation of fibrosis-associated proteins in the cord blood of the neonate. These fibrosis-associated molecules are associated with adverse birth outcomes such as low birth weight (LBW), small for gestational age and prematurity [16]. In addition, endotoxin levels in both maternal and placental compartments in pregnant women with schistosomiasis are 1.3- and 2.4-folds higher, respectively, compared to uninfected women. This higher concentration of endotoxin in placental blood is associated with preterm birth, acute chorioamnionitis, and elevated proinflammatory cytokines [17].

## 3. Control of Schistosomiasis in the Philippines

In the 1980s, when the highly effective anti-schistosome drug praziquantel (PZQ) was introduced in the Philippines, the schistosomiasis control program rolled out a large-scale community-based chemotherapy approach to eliminate the risk of parasite-associated morbidity – this approach became the backbone of schistosomiasis control in the Philippines [18]. Other control measures to prevent transmission from snail intermediate hosts to humans like health education, behavioural modification, improved sanitation and snail control were continued but not sustained and only on a limited scale. After more than three decades of community-based chemotherapy with PZQ, challenges with this approach have surfaced [18]. Extensive community-based campaigns including mass drug administration (MDA) in the last 10 years has reduced the parasite-associated clinically apparent morbidities, although the hepato-splenic form of schistosomiasis japonica persists in hard to reach endemic zones. Subtle or subclinical morbidities like growth retardation and anaemia in schistosome infected children and poor outcomes of pregnancy in infected women still persist in endemic areas [18,19]. Community-based chemotherapy also failed to interrupt parasite transmission [18,19].

## 4. Innovative Approach towards Elimination of Schistosomiasis in the Philippines

Despite the limitations of current control measures, it is certain that drug delivery through MDA will be continued for an indefinite period for the control of schistosomiasis in the country. Relaxation of the MDA program or development of parasite resistance to PZQ will result in rebound of schistosome-induced infection and disease [20]. However, while PZQ remains highly effective, additional control measures should be added to augment the community-based chemotherapy control program and move beyond just morbidity control [21]. The next step is elimination of reinfection to prevent all forms of schistosome-induced morbidities. This step will require a comprehensive and more effective phase of disease control and will require incremental expense, but these would ultimately be offset by the greater health benefits achieved with complete elimination of parasite transmission.

Two measures can markedly improve the control program for schistosomiasis in the Philippines. These measures include: (1) Improving delivery or coverage of PZQ to 85% through an intensive MDA program and yearly treatment of 85% of the bovines [22]. Increasing compliance to MDA in humans from less than 50% to 85% is doable. However, yearly treatment of bovines will be very difficult to sustain. (2) MDA (85% coverage) plus removal of water buffaloes (carabaos) from endemic areas and replacing these animals with mechanized tractors. Mechanized farming will prevent exposure of the farmers to the parasite during the tilling of the rice field and planting and harvesting of rice. This approach has proven successful in China where removing buffalos in endemic areas can profoundly reduce the transmission of *S. japonicum* to humans by 75 to >90% [23]. In the Philippines, recent consultations with the Local Government Unit (LGU) in the Municipality of Javier, a schistosomiasis-endemic area in the Province of Leyte, demonstrated the willingness of the farmers to replace the carabaos with mechanized tractors. In their experience replacing of carabaos by tractors will improve the income of the people in the endemic areas because the cost of labour for rice farming will be markedly reduced and the frequency and volume of rice harvest per year will increase. In addition, they also noted that if they plant hybrid instead of inbred rice the volume of rice recovered per harvest would be significantly increased. Disease transmission from cattle that are also in the endemic areas can be prevented by treating the animals with PZQ—this is would be more sustainable than treating all bovines due to the reduced numbers. This strategy should also include other measures that are doable in the endemic villages like health education and behavioural modification. Recently, mechanization of rice farming is being implemented at pilot scale by a private company in the town of Alang Alang, a schistosomiasis endemic village in the Province of Leyte. In this project it has been demonstrated that the income of farmers can be increased up to ten times compared to their income using the traditional rice farming method [24]. However, the success of this project needs validation in typical endemic areas where resources are limited. It is also important to know by regular monitoring by the Department Health whether the outcome in terms of reduction of *S. japonicum* infection in humans will be significant and sustainable.

Vaccines may also play a crucial role in the elimination of schistosomiasis in the Philippines. No human vaccine is currently available but some veterinary-based transmission blocking vaccines targeting bovines do show some promise and are undergoing field trials [25,26] An alternative elimination strategy would therefore combine human treatment (85% coverage) and treatment of bovines (85% coverage), followed by immunization of these animals with a transmission blocking anti-schistosome vaccine.

## 5. Conclusions

A multi-component integrated approach towards the control of *S. japonicum* in the Philippines is critical for long-term sustainable control and eventual elimination. Key to such an approach is ensuring high PZQ coverage in endemic populations and the targeting of bovines (carabaos) through either their removal and replacement with mechanized tractors in endemic areas or vaccination.
